# Sensory Ataxic Neuropathy in Golden Retriever Dogs Is Caused by a Deletion in the Mitochondrial *tRNA^Tyr^* Gene

**DOI:** 10.1371/journal.pgen.1000499

**Published:** 2009-05-29

**Authors:** Izabella Baranowska, Karin Hultin Jäderlund, Inger Nennesmo, Erik Holmqvist, Nadja Heidrich, Nils-Göran Larsson, Göran Andersson, E. Gerhart H. Wagner, Åke Hedhammar, Rolf Wibom, Leif Andersson

**Affiliations:** 1 Department of Animal Breeding and Genetics, Swedish University of Agricultural Sciences, Uppsala, Sweden; 2 Department of Clinical Sciences, Swedish University of Agricultural Sciences, Uppsala, Sweden; 3 Department of Companion Animal Clinical Sciences, Norwegian School of Veterinary Science, Oslo, Norway; 4 Department of Laboratory Medicine, Karolinska Institutet, Stockholm, Sweden; 5 Department of Cell and Molecular Biology, Uppsala University, Uppsala, Sweden; 6 Department of Medical Biochemistry and Microbiology, Uppsala University, Uppsala, Sweden; University of Liège, Belgium

## Abstract

Sensory ataxic neuropathy (SAN) is a recently identified neurological disorder in golden retrievers. Pedigree analysis revealed that all affected dogs belong to one maternal lineage, and a statistical analysis showed that the disorder has a mitochondrial origin. A one base pair deletion in the mitochondrial *tRNA^Tyr^* gene was identified at position 5304 in affected dogs after re-sequencing the complete mitochondrial genome of seven individuals. The deletion was not found among dogs representing 18 different breeds or in six wolves, ruling out this as a common polymorphism. The mutation could be traced back to a common ancestor of all affected dogs that lived in the 1970s. We used a quantitative oligonucleotide ligation assay to establish the degree of heteroplasmy in blood and tissue samples from affected dogs and controls. Affected dogs and their first to fourth degree relatives had 0–11% wild-type (wt) sequence, while more distant relatives ranged between 5% and 60% wt sequence and all unrelated golden retrievers had 100% wt sequence. Northern blot analysis showed that *tRNA^Tyr^* had a 10-fold lower steady-state level in affected dogs compared with controls. Four out of five affected dogs showed decreases in mitochondrial ATP production rates and respiratory chain enzyme activities together with morphological alterations in muscle tissue, resembling the changes reported in human mitochondrial pathology. Altogether, these results provide conclusive evidence that the deletion in the mitochondrial *tRNA^Tyr^* gene is the causative mutation for SAN.

## Author Summary

Mitochondrial disorders are a group of heterogeneous diseases. It has been estimated that the prevalence of mitochondrial diseases in humans, due to mutations of the mitochondrial genome (mtDNA), is approximately 1 in 8000 in a Caucasian population. Since the late 1980s, when the first disease-causing mutation in human mtDNA was identified, approximately 250 pathogenic mtDNA mutations have been described. Sensory ataxic neuropathy (SAN) is a recently identified neurological disorder in golden retriever dogs that is maternally transmitted. Affected dogs are ataxic, have postural reaction deficits, and exhibit reduced spinal reflexes. They have no pronounced muscle atrophy nor do they seem to be in pain. In this study, we report the identification and characterization of the mutation causing SAN, a single base pair deletion in the mitochondrial *tRNA^Tyr^* gene. The identification of this mutation makes it possible to eradicate the disease in golden retrievers. SAN constitutes a new animal model for mitochondrial disorders in humans.

## Introduction

Sensory ataxic neuropathy (SAN) is a recently identified neurological disorder in golden retrievers [1]. SAN has an insidious onset during puppyhood, followed by slow progression. Males and females are affected at similar frequencies. Affected dogs are ataxic, have postural reaction deficits and reduced or absent spinal reflexes. They have no pronounced muscle atrophy, and the dogs do not seem to be in pain. Electrophysiological examination revealed that they have reduced conduction velocities of nerve impulses in sensory nerves. Pathological examination indicated degenerative changes both in the central and peripheral nervous system. Approximately fifty percent of the affected dogs were euthanized before three years of age. A preliminary examination of pedigree data showed that all affected dogs could be traced back to a female on the maternal side that lived in the 1970s, suggesting that SAN could be caused by a mutation in the mitochondrial genome (mtDNA).

Mitochondrial disorders, caused by mutations in maternally inherited mtDNA, are a group of heterogeneous diseases in humans. More than 250 pathogenic point mutations as well as small and large scale rearrangements of mtDNA have been identified [2], and with an estimated incidence of approximately 1 in 8000 in the Caucasian population, mitochondrial disorders are considered to be among the most common forms of metabolic disease [3]. They usually manifest in energy-consuming tissues such as the central nervous system, muscles, auditory system and visual system, but almost any organ in any combination might be involved and age at onset often varies widely.

The genotype/phenotype relationship for mtDNA mutation diseases is only partly understood. A somatic mammalian cell typically contains thousands of mtDNA molecules. Typically only one type of mtDNA is present in a cell, a condition referred to as homoplasmy. In mitochondrial diseases and in ageing a mixture of wild-type and mutated mtDNA may be simultaneously present in a cell, a condition referred to as heteroplasmy [4]–[6]. Mutated mtDNA will only impair respiratory chain function if it is present at a minimal threshold level. Furthermore, the relative proportion of wild-type and mutated mtDNA may vary widely between tissues in an individual. The disease manifestation in these disorders will thus be determined by a complex interplay between mtDNA (mutation levels and distribution) and the nuclear genetic background. A well-known example of the interplay between mutated mtDNA and nuclear genes is Leber's hereditary optic neuropathy (LHON), where the reduced penetrance of the phenotype is influenced by nuclear loci [7].

Two thirds of disease-causing mtDNA mutations occur in *tRNA* genes, which only constitute approximately 9% of the mitochondrial genome. This suggests that *tRNA* genes are “hotspots” for mitochondrial mutation [8],[9]. Experimental studies in the mouse supports this idea and has shown that *tRNA* gene mutations are much more likely to be maternally transmitted than mutations in protein coding genes [10]. The *tRNA* mutations typically are heteroplasmic and affect mitochondrial translation, thereby causing reduced expression of all mtDNA-encoded proteins. However, impaired translation and respiratory chain deficiency will only occur if the level of mutated mtDNA exceeds a certain threshold.

Several animal models are available that mimic the pathologic state of mitochondrial disorders in humans [11],[12]. Very few of them do however resemble a natural situation that allows the inheritance of a single *tRNA* mutation to be studied over several generations. We here show that SAN in golden retrievers is caused by a mutation in the mitochondrial *tRNA^Tyr^* gene. Thanks to a detailed breeding registry, we were able to trace the mutation more than 10 generations back in time.

## Results

### Pedigree Analysis and Re-Sequencing

**Figure 1:**
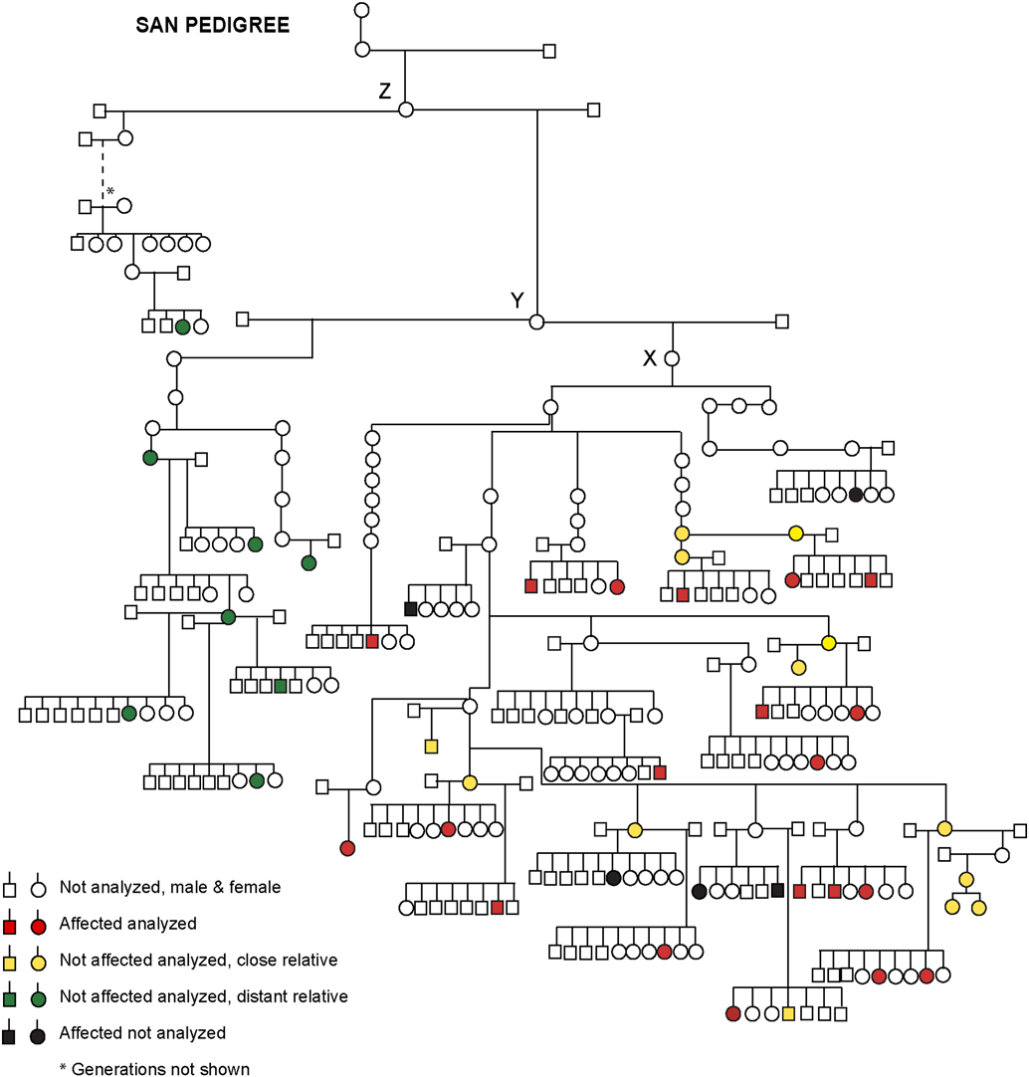
A schematic pedigree of golden retrievers affected by sensory ataxic neuropathy (SAN) and their relatives. In total we had access to blood samples from 20 out of 25 affected dogs (marked red or black) that can be traced back on the maternal side to female X. We also had access to samples from 13 first, second, third or fourth degree relatives of affected dogs (marked yellow). More distant relatives descendant of females Y or Z are marked with green. Only litters with affected dogs and sampled litters are included.

Pedigree analysis of the 25 affected dogs revealed that they all belong to the same maternal lineage, and can be traced back to a female X that lived during the 1970s (Figure 1). In order to test if these data exclude a possible nuclear inheritance we calculated the prevalence of disease in offspring from all males and females in those litters where the mothers to the affected dogs were one of the siblings. If the risk factor was present in the nuclear genome, the proportion of affected progeny should be about the same from male and female parents in this maternal lineage. By contrast, all cases should derive from female parents if the risk factor is encoded in the mtDNA genome. We observed 25 affected dogs among 272 progeny from female dogs but no affected dogs among 177 progeny sired by males (P<10^−6^; Fisher's exact test). This provides conclusive evidence that SAN shows mitochondrial transmission.

**Figure 2:**
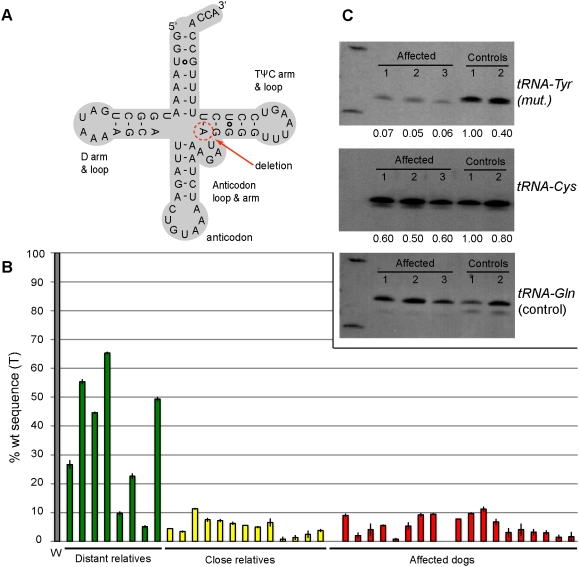
Characterization of the *ΔT5304* deletion in the *tRNA^Tyr^* gene. (A) The mutation deleted the A of the innermost base pair of the TΨC stem, as indicated in the cloverleaf structure of *tRNA^Tyr^*. (B) A quantitative oligonucleotide ligation assay (qOLA) was used to estimate the relation between wild-type and mutant molecules in blood samples from golden retrievers. W represents 71 unrelated golden retriever dogs that all show 100% wt molecules. The green bars represent the degree of heteroplasmy of distant relatives (34.8 (22.0); mean (SD)), yellow bars stand for close relatives (5.1 (2.8)) and the red bars represent affected dogs (4.9 (3.5)). (C) Northern blot analyses using probes for mt *tRNA^Tyr^*, mt *tRNA^Cys^* and mt *tRNA^Gln^* and RNA from three SAN-affected golden retrievers and two unaffected dogs; one dachshund (Control 1) and one golden retriever (Control 2). The values given at the bottom of the blots for mt *tRNA^Tyr^* and mt *tRNA^Cys^* is the relative hybridization intensity normalized using the hybridization intensity for mt *tRNA^Gln^*. The sample with the highest normalized hybridization intensity was given the value 1.00.

We re-sequenced the complete mitochondrial genome of seven dogs: four affected golden retrievers (GRs), one close relative and two unrelated GRs. A one base pair deletion was identified at position 5304 in *tRNA^Tyr^* of the affected dogs and their relatives (Figure 2A). This was the only sequence variant that was uniquely associated with the disease. This variant has not been found in any other dog breed and this position in mtDNA is highly conserved among vertebrates.

### Assessment of Heteroplasmy

We evaluated two different methods, pyrosequencing and quantitative oligonucleotide ligation assay (qOLA), to quantify the level of heteroplasmy of the deletion in the *tRNA^Tyr^* gene (Text S1; Figure S1). The qOLA method provided superior resolution and was therefore applied (Figure 2B). We had access to blood samples from 20 out of the 25 affected golden retrievers, and from 13 first, second, third or fourth degree relatives that did not display the clinical disease phenotype. Additional more distant relatives (n = 8) descendant from female Z were also analyzed (Figure 1). We were unable to collect any blood samples tracing further back than to female Z because these dogs are either dead or the registry is incomplete. We also searched for the deletion in 71 healthy unrelated golden retrievers, in 86 dogs representing 18 other breeds and in six wolfs. All unrelated dogs had 100% wt sequence, whereas among the distant relatives (tracing back to female Z, but not to female X; Figure 1) we observed a large variation in mutation load, ranging from 5–60% wt, indicating that female X that we initially assumed to be the founder was in fact not. The affected individuals had a very low level of wt sequence (0–11.2%). Low levels of wt sequences were also found among clinically healthy close relatives, and the proportion of % wt sequence overlapped between the two groups (Figure 2B). There was no strong correlation between the degree of heteroplasmy and the degree of relatedness among close relatives. Thus, this very sensitive test did not unambiguously distinguish affected individuals from their close relatives that showed no clinical signs of disease when analyzed in blood.

We also analyzed the degree of heteroplasmy in different tissues from three affected dogs. The frontal lobe, spinal cord with dorsal root ganglia (7^th^ thoracal and 5^th^ lumbar), optic nerve, recurrent laryngeal nerve, pancreas, thyroid gland, and skeletal muscle of both pelvic and thoracic limb were analyzed by qOLA. The results revealed that all analyzed tissues had a higher mutation load, close to 0% wt, compared with that found in blood cells from the same affected individuals. Unfortunately, we do not have access to tissues from clinically healthy relatives with a similarly high mutation load as that found in the affected dogs.

### Northern Blot Analysis

Steady-state levels of three mtDNA *tRNA* species were assessed by northern blot analysis. Analysis of *tRNAs* extracted from muscle tissue of three SAN-affected dogs and two controls, a dachshund and a healthy golden retriever, showed that all three SAN-affected dogs had significantly reduced levels of *tRNA^Tyr^* compared to the two controls (Figure 2C); the hybridization intensity was on average about 10-fold lower than for controls (P<0.001 in a student's t-test). In contrast, all affected dogs showed normal expression of *tRNA^Cys^* and *tRNA^Gln^* compared with the controls (Figure 2C).

### Structure Probing

*In vitro* transcribed wt and mutant *tRNA^Tyr^* was radioactively labeled at the 5′-end and subjected to partial digestion using either lead (Pb^2+^) or RNase T1 (Figure S2). The cleavage patterns did not reveal any significant structural differences between the two molecules.

### Mitochondrial ATP-Production Rate and Respiratory Chain Enzyme Activity

**Figure 3:**
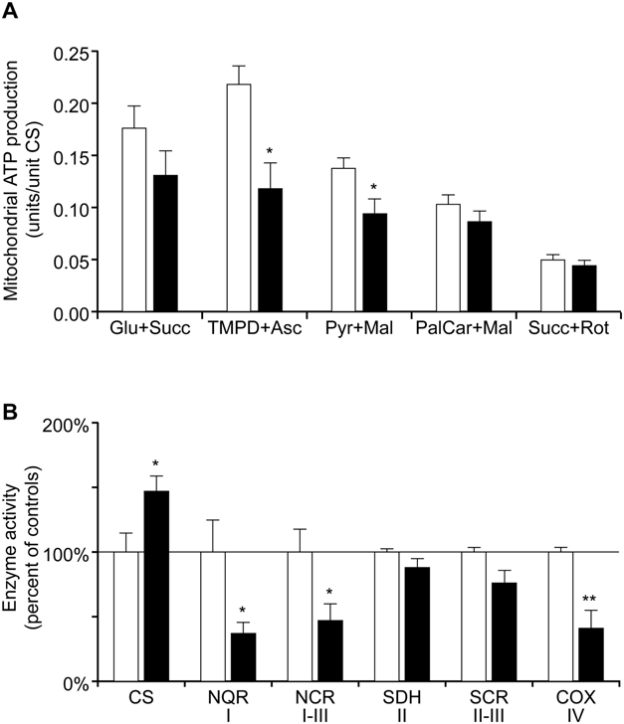
Mitochondrial ATP production and respiratory chain enzyme activity. Biochemical analyses of respiratory chain function in affected (filled bars) and control (open bars) animals. Bars represent mean levels ±SEM. n = 5 in both groups. *, P<0.05; **, P<0.01. (A) Mitochondrial ATP production rate in the presence of the substrate combinations glutamate+succinate, TMPD+ascorbate, pyruvate+malate, palmitoyl-L-carnitine+malate and succinate+rotenone, which enter the respiratory chain at different points. (B) Relative activities of citrate synthase (CS) in muscle tissue and respiratory chain enzymes in isolated mitochondria. NADH coenzyme Q reductase (NQR), corresponding to complex I; NADH cytochrome c reductase (NCR), complex I–III; succinate dehydrogenase (SDH), complex II; succinate cytochrome c reductase (SCR), complex II–III; cytochrome c oxidase (COX), complex IV. The relative enzyme activities presented as 100% in the figure correspond to the following absolute values: CS 61 mmol/min/kg muscle, NQR 0.13, NCR 1.4, SDH 0.36, SCR 0.76 and COX 2.8 units/unit CS. Raw data for individual dogs are given in Table S1.

We found a decreased rate of ATP production in isolated muscle mitochondria from affected animals (Figure 3A; Table S1). This decrease was most pronounced with the substrate combinations N,N,N^1^,N^1^-tetramethyl-1,4-phenyldiamine (TMPD)+ascorbate (−46%) and pyruvate+malate (−32%). TMPD+ascorbate is an artificial electron carrier that acts directly on Complex IV in the respiratory chain, thus selectively measuring the ATP producing capacity in Complex IV and V. ATP production from pyruvate+malate involves all enzymes in Krebs cycle and the respiratory chain. Maximal endogenous ATP production, obtained with glutamate+succinate, was not significantly decreased. Measurement of the activities of different respiratory chain enzyme complexes showed decreased activities of complex I (−63%), complex I–III (−53%) and complex IV (−59%) in the affected dogs in comparison with healthy controls (Figure 3B; Table S1). The two measurements involving Complex II did not show significantly altered activities. Measurements of the enzyme activities of respiratory chain complexes and the ATP production rate thus gave consistent results indicating dysfunction of complex I and IV, which are dependent on subunits encoded by mitochondrial DNA for their function. The function of complex II is only dependent on nuclear genes and was normal. There was an elevation of citrate synthase activity in the muscle tissue (+47%) (Figure 3B) consistent with a mitochondrial proliferation often observed in respiratory chain deficient tissues.

One of the five affected animals that contributed to the data presented in Figure 3 did neither show decreases in ATP production nor in respiratory chain enzyme activities, and was therefore not possible to distinguish from the healthy controls with these assays.

### Histochemistry and Electron Microscopy of Muscle Fibers

**Figure 4:**
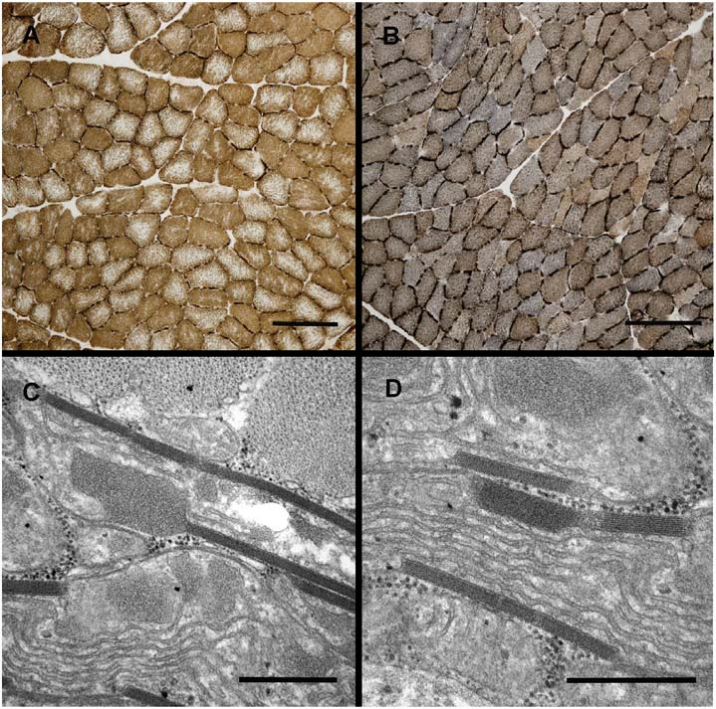
Histochemistry and electron microscopy of muscle fibers from SAN-affected (B, C and D) and control dogs (A). (A and B) Histochemistry showing the combined reaction for SDH and COX. Note the bluish reaction for the affected dog, indicating lack of COX activity. Bar = 200 µm. (C and D) Electron microscopy showing paracrystalline inclusions in a case. Bar = 0.5 µm.

Hematoxylin-eosin staining showed normal morphology for all examined biopsies. For the oxidative enzymes the reactions were more even and compact over the cut surface of the fibers for four of the affected dogs and in one control healthy dog (Table S1). In the combined reaction for succinate dehydrogenase (SDH) and cytochrome oxidase (COX) these also showed a bluish staining compared with the controls and affected dog nr. 3 (Figures 4A and B). Fibers showing total lack of cytochrome oxidase activity were not detected.

Electron microscopy revealed only clear signs of mitochondrial pathology for one of the dogs (affected dog nr. 3), which in light microscopy however did not show the bluish staining in the combined SDH/COX reaction. Paracrystalline inclusions were present in some of the fibers of this dog (Figures 4C and D).

## Discussion

The pedigree analysis provided conclusive evidence that SAN is a maternally inherited disorder. Re-sequencing of the complete mtDNA revealed a one base pair deletion in the mt *tRNA^Tyr^* gene in a region that is conserved across mammalian species. A qOLA assay of the proportion of mutant and wild-type mtDNA showed that the different groups of dogs carry different mutation load, however the mutation load in blood of affected dogs and their close relatives overlap. The lack of a perfect correlation between our estimate of heteroplasmy and the clinical phenotype is most likely due to the fact that the disease is manifested in the CNS while we measured the mutation load in blood cells. It is well known that the nuclear genetic background can affect the penetrance of pathogenic mtDNA mutations and it can therefore not be ruled out that nuclear polymorphisms as well as unidentified environmental factors may contribute to the risk of disease manifestation.

More distant relatives (tracing back to female Y and Z) showed a larger variation in mutation load and a higher fraction of wild-type copies compared to dogs traced back to female X (Figure 2B). The fact that disease only occurred in the branch of the pedigree with the highest proportion of the one base-pair deletion in *tRNA^Tyr^* supports that this is the causative mutation.

Northern blot analysis demonstrated lower steady-state levels of *tRNA^Tyr^* in muscle tissue from affected dogs, compared to healthy controls. This can either be explained by a processing defect impairing the formation of the mature *tRNA^Tyr^* or by an increased turnover of the mutant *tRNA^Tyr^*. We can refute the former option. Firstly, northern blots with probes detecting *tRNA^Tyr^* showed no additional high molecular weight RNAs indicative of aberrant processing. Secondly, *tRNA^Tyr^* and *tRNA^Cys^* are transcribed as a polycistronic precursor and a processing defect should affect both molecules. The fact that the steady state level of *tRNA^Cys^* appears unaffected argues strongly against an aberrant processing. Thus, our data indicate that the deletion impairs the stability of the mutated *tRNA^Tyr^*.

Although the affected animals did not show any obvious overt clinical signs in muscle, a severely affected muscle mitochondrial function was observed in a majority of the examined individuals. The decreases in mitochondrial ATP production and in the respiratory chain activities of complex I and IV are all findings consistent with alterations found in humans and mice affected by mtDNA disorders [11], [13]–[15]. Increased citrate synthase activity is frequently observed in mitochondrial disorders and is thought to reflect mitochondrial proliferation in response to energy deficit in the diseased tissue [12],[15]. One of the affected dogs had nearly normal respiratory chain enzyme activities, possibly due to a mosaic tissue distribution of the heteroplasmy [16].

The disturbed muscle mitochondrial function was accompanied by a divergent pattern in the combined histochemical staining for cytochrome c oxidase (COX) and succinate dehydrogenase (SDH). The affected animals showed a bluish staining, indicating a shift towards a lower COX activity compared to the SDH activity. Fibers totally lacking COX-activity and ragged red fibers are common findings in humans harboring large scale deletions and *tRNA* mutations in mtDNA [13],[14],[17]. Strikingly, no such fibers were found in muscle samples from the dogs. A possible explanation for this observation and the fact that SAN-affected dogs did not have any clinical overt signs of muscle disease, might be the different muscle composition in dogs, with a higher mitochondrial density compared to humans [18]. Despite the lack of ragged red fibers, mitochondrial paracrystalline inclusions were found in one of the animals, a characteristic finding in human adult mitochondrial myopathies [17]. Since dogs have been euthanized before they have acquired life-threatening clinical signs, the end stage pathology of the disease is unknown.

According to DiMauro & Davidzon (2005) [19] a mitochondrial mutation is likely to be pathogenic if 1) the mutation is not present in healthy normal individuals, 2) it changes an evolutionarily conserved site, 3) the mutation causes a respiratory chain enzyme deficiency in affected tissues or defects in mitochondrial protein synthesis and respiration demonstrable in cybrid cell lines, and 4) a correlation between heteroplasmy and clinical phenotype is observed. The deletion reported in this study fulfills all the criteria listed above, except for the first one, as we observed clinically healthy, close relatives with high mutation load. However, some exceptional cases of pathogenic mutations that are homoplasmic in non-affected humans have also been reported [6],[20]. Furthermore, all cases in the present occurred in the part of the pedigree with the highest mutation load and all unrelated dogs were 100% wild-type for position 5304 in *tRNA^Tyr^*. Thus, this study has provided conclusive evidence that SAN is a mitochondrial disorder and that *ΔT5304* in *tRNA^Tyr^* is the causative mutation.

This is the second report of an mtDNA disorder in dogs. In a previous study, Li et al. (2006) identified a mutation in the mitochondrial *cytochrome b* gene that is associated with encephalomyelopathy in families of Shetland sheepdogs and Australian cattle dogs [21]. Other studies reporting suspected mitochondrial disorders in dogs (*i.e.* mitochondrial myopathies or encephalopathies) are based on single or few cases and are restricted to clinical, pathological and biochemical aspects of the disorders, thus not including complete or partial mtDNA sequencing to identify the disease-causing mutation [22]–[24].

In humans, mutations in the mitochondrial *tRNA^Tyr^* gene have been associated with mitochondrial disorders [25]–[28]. These cases involve mutations at other positions (*A5843G*, *A5874G*, *G5877A* and *ΔT5885*,) than the one associated with SAN in dogs (*ΔT5304*), which corresponds to position 5848 in humans. Sahashi et al. [25] and Raffelsberger et al. [26] described two female patients with CPEO (chronic progressive external ophthalmoplegia) with age of onset at 28 and 41 years of age and exercise intolerance, whereas Scaglia et al. [27] reported a female patient with mitochondrial cytopathy progressing to focal segmental glomerulosclerosis and cardiomyopathy. Similar clinical signs have not been observed in the golden retrievers reported in this study.

This study documents one of the most extensive pedigrees with a mtDNA disorder in any species. We identified 25 affected dogs and traced the mutation back at least to female Z (Figure 1), born in 1971. It has been transmitted silently since then, and the first case of an affected dog identified by us was born 1997. Based on information from the studbook we estimate that about 5% of female dogs in the Swedish golden retriever population born 2001–2005 carry this mutation. The prevalence in other countries is at present unknown because we were not able to trace the mutation further back than to female Z. The most interesting aspect of this study is the mild phenotypic effect of the mutation which allows very high mutation loads (down to almost 0% wild-type sequence in blood cells) in clinically healthy individuals. It therefore constitutes an interesting model for human mtDNA disorders that may be used for testing therapeutic approaches for treating mitochondrial disorders.

## Materials and Methods

### Animal Tissues

Blood and tissue samples were collected from privately owned dogs with the owners written consent. The extracted DNA (QIAamp DNA Blood Mini Kit, Qiagen, Valencia, CA) was kept at −20°C until it was used. Tissue samples were collected in immediate conjunction with euthanasia, at the Swedish National Veterinary Institute, Uppsala, Sweden, and were kept at −80°C prior to use. Fresh muscle biopsies for functional studies, electronmicroscopy and histopathology were collected from *m. quadriceps vastus medialis* of five affected dogs (1–5 years of age) and five age-matched control GR dogs under sedation and local anesthesia. The study was approved by Uppsala Animal Ethics Committee.

### Pedigree Analysis

The pedigree was analyzed using information of relatedness from the Swedish Kennel Club registry (www.skk.se).

### mtDNA Re-Sequencing

The complete mitochondrial genome was re-sequenced from fifteen overlapping amplicons. All PCR primers generated amplicons ranging from approximately 1200–1500 bp and were designed using the Primer3 software [29]. The PCR primers were reused for sequencing together with four additional internal sequencing primers per amplicon (Table S2). Contigs were aligned and analyzed using Codon Code (Dedham, MA). Complete mitochondrial sequences of four affected dogs (GenBank FJ817358, FJ817359, FJ817360, FJ817361), one close relative (FJ817362) and two unrelated golden retrievers (FJ817363, FJ817364) have been deposited in GenBank. The numbering of nucleotide positions refers to the complete dog mtDNA sequence with GenBank accession number NC_002008.

### Quantification of *tRNA^Tyr^* Deletion

Quantitative oligonucleotide ligation assay (qOLA) method was used to estimate the mutation load in blood and tissue samples, but also for evaluation purposes. Each sample was PCR-amplified according to standard procedure using the primers listed in Table S3. PCR products were treated with proteinase K (20 ng/ul) at 98°C for 1 h and then used for a ligation reaction containing 1×Reaction buffer, 1 pmol F-primer, 0.5 pmol R1-primer, 0.5 pmol R2-primer, 1.5 U Ampligase Epicentre Biotechnologies, Madison, WI) at 94°C 10 min, 10 cycles at 94°C 30 s and 50°C 1.5 min. The ligation products were separated using capillary gel electrophoresis on a MegaBACE (GE Healthcare, Uppsala, Sweden) and analyzed using MegaBACE Fragment Profiler Version 1.2. All blood samples, except two, were analyzed in triplicates. Dogs representing the following 18 breeds were also analyzed for the presence of the mutation: hovawarts (n = 5), giant schnauzers (n = 4), dachshunds (n = 7), Nova Scotia duck tolling retrievers (n = 5), boxers (n = 5), fox terriers (n = 4), Dalmatians (n = 4), basenjis (n = 4), Bernese mountain dogs (n = 4), border collies (n = 4), bullterriers (n = 5), beagles (n = 5), shar peis (n = 5), poodles (n = 5), bearded collies (n = 5), Irish wolfhounds (n = 5), German shepherds (n = 5) and West Highland white terriers (n = 5).

### Northern Blot

Total RNA was extracted using the mirVana miRNA Isolation Kit (Ambion, Austin, TX). One µg of RNA was denatured in formamide loading dye (FD; 90% formamide, 15 mM EDTA, 0.05% bromophenol blue, 0.05% xylene cyanol), boiled, loaded and run on a 12.5% denaturing polyacrylamide gel. A labeled pUC19 RNA ladder (Fermentas, Burlington, Canada) was used as size marker. The RNA was electroblotted onto Nylon N+ membranes (GE Healthcare) at 10 V for 15 h. Transfer was performed in a Biorad blotting chamber in 1×TBE buffer at 4°C. Prehybridization was done for 2–4 h at 60°C in 15 ml prehybridization buffer (5× SSC, 100× Denhardt, 50 mM sodium phosphate pH 6.7, 1% dextran sulphate, 0.1% SDS) with 75 µl Herring sperm DNA (20 mg/ml). Hybridization was performed o/n at 60°C in the same buffer lacking Herring sperm DNA but containing labeled oligonucleotide probe. Probes were labeled (40 pmol probe, 10× kinase buffer, T4 polynucleotide kinase (PNK, Ambion, Austin, TX), [γ-^32^P]ATP) at 37°C for 45 min. Prior to hybridization, the labeled probe was run through a G-50 column (GE, Healthcare, Uppsala, Sweden). Probe sequences are listed in Table S4. Membranes were washed once for 20 min at 60°C in 2× SSC, 0.5% SDS, and once for 20 min in 0.5× SSC, 0.5% SDS. Signals were detected and quantified using a Molecular Dynamics PhosphorImager model 400S (Sunnyvale, CA) and ImageQuant software version 4.2a. The hybridization intensities of *tRNA^Tyr^* and *tRNA^Cys^* were normalized using the hybridization intensity obtained for *tRNA^Gln^* from the same dog.

### Structure Probing

PCR-amplified products of one affected and one unrelated GR were cloned using the TOPO TA Cloning kit (Invitrogen, Carlsbad, CA). The forward primer contained a T7 RNA polymerase promoter sequence tag to generate *in vitro* transcription templates (Table S5). Sequence validated PCR-products (wt or mutant) were used in *in vitro* transcription reaction NTPs (25 mM), 4× Trx buffer, RNA guard, T7 RNA polymerase (200 U/µl, Ambion, Austin, TX) at 37°C for 4 h. RQ1 DNAse was added (37°C at 30 min) to remove the DNA template, followed by phenol/chloroform extraction and EtOH precipitation. The RNA was spun through a G-50 column (GE Healthcare, Uppsala, Sweden) and dephosphorylated (40 pmol RNA, 10× shrimp alkaline phosphatase [SAP] buffer, and SAP at 37°C, 1 h). After an additional phenol/chloroform treatment and precipitation, redissolved RNA was 5′-end labeled by treatment with polynucleotide kinase (40 pmol RNA, 10× kinase buffer, PNK, [γ-^32^P]ATP at 37°C for 1 h) and separated on a 8% denaturing polyacrylamide (PA) gel (1 µg of RNA/lane).

After short exposure to X-ray film, the RNA-containing gel slices were excised and eluted by shaking o/n in RNA elution buffer (0.1 M sodium acetate pH 5.6, 10 mM EDTA, 0.5% SDS). Following phenol/chloroform extraction and EtOH precipitation, one µl aliquots of the redissolved RNAs (wt and mutant) were used for preparation of alkaline ladders (OH-ladder), T1-ladder, Control (untreated), or for partial digestion under native conditions by RNase T1 (0.01 U, Ambion, Austin, TX) or Pb^2+^ (25 mM) according to Table S6. Reactions and subsequent steps were carried out as described [30]. The RNA samples were run on a 12% PA-gel (40 W, 1.5 h), followed by transfer to a filter paper, vacuum dried (SGD5040 Slab Gel Dryer, ArtisanScientific, Champaign, IL), and analyzed by PhosphorImager as above.

### Mitochondrial ATP-Production Rate and Respiratory Chain Enzyme Activity

Mitochondria were isolated from the fresh muscle tissue, and respiratory chain enzyme activities and mitochondrial ATP production rates (MAPR) were determined blindly as described [31]. Citrate synthase activity in muscle tissue and in isolated mitochondria was determined according to the same paper.

### Histochemistry, Light, and Electron Microscopy

The muscle biopsies were rapidly frozen in 2-Methylbutane/ice. Eight µm thick cryosections were stained with hematoxylin-eosin and Gomori trichrome as well as for oxidative enzymes (NADH-tetrazolium reductase, succinate dehydrogenase, cytochrome oxidase and the combined reaction for succinate dehydrogenase and cytochrome oxidase). Pairs of biopsies (one case and one control) were stained at the same occasion. Muscle samples were also fixed in 2.5% glutaraldehyde for electron microscopy.

## Supporting Information

Figure S1 Evaluation of quantification methods: pyrosequencing and quantitative oligonucleotide ligation assay (qOLA). (A) Pyrosequencing of wide dilution series. (B) Pyrosequencing of narrow dilution series. (C) qOLA of wide dilution series. (D) qOLA of wide dilution series. By comparing the opposite extremes (0 and 100%) of dilution series of Fig. S1 A and C, it is apparent that qOLA gives a more accurate estimate of these values. This is further confirmed by comparing Figure S1B and D, where the linearity is obvious in D, but not in B.
(0.25 MB TIF)

Figure S2 Structure probing of *tRNA^Tyr^*. Transfer *RNA^Tyr^* was in vitro transcribed, labeled and cleaved with RNase T1 or Pb^2+^. By comparing lanes from wt and mutant there is no obvious alteration in the structure of the mutant *tRNA^Tyr^*.
(4.46 MB TIF)

Table S1 Overview of heteroplasmy in blood and muscle, and morphological and biochemical examination in the five dog pairs tested.
(0.05 MB DOC)

Table S2 PCR and sequencing primers.
(0.10 MB DOC)

Table S3 Quantification primers.
(0.03 MB DOC)

Table S4 Northern probes.
(0.02 MB DOC)

Table S5 Structure probing sequences.
(0.03 MB DOC)

Table S6 Preparation of OH-ladder, T1-ladder, Control, T1 (0.01 U), Pb^2+^ (25 mM).
(0.04 MB DOC)

Text S1 Evaluation of quantification methods - pyrosequencing and quantitative oligonucleotide ligation assay.
(0.02 MB DOC)
