# Supplementary figures and images for: Sensory Ataxic Neuropathy in Golden Retriever Dogs Is Caused by a Deletion in the Mitochondrial tRNATyr Gene
Source: PLoS Genet. 2009 May 29;5(5):e1000499. doi: 10.1371/journal.pgen.1000499 (PMC2683749; doi:10.1371/journal.pgen.1000499)

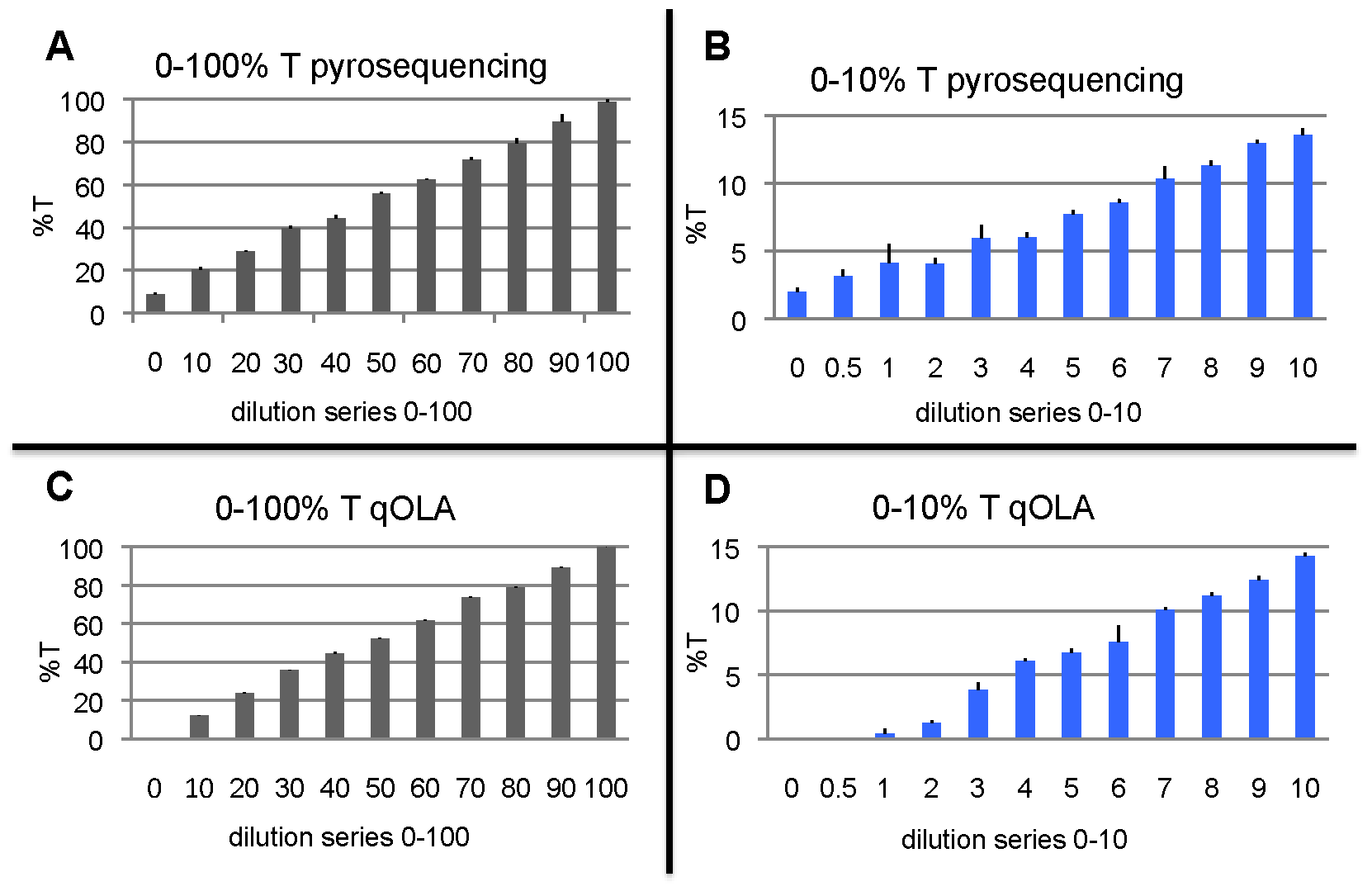

Supplement: Figure S1 — Evaluation of quantification methods: pyrosequencing and quantitative oligonucleotide ligation assay (qOLA). (A) Pyrosequencing of wide dilution series. (B) Pyrosequencing of narrow dilution series. (C) qOLA of wide dilution series. (D) qOLA of wide dilution series. By comparing the opposite extremes (0 and 100%) of dilution series of Fig. S1 A and C, it is apparent that qOLA gives a more accurate estimate of these values. This is further confirmed by comparing Figure S1B and D, where the linearity is obvious in D, but not in B. (0.25 MB TIF) [file pgen.1000499.s001.tif]

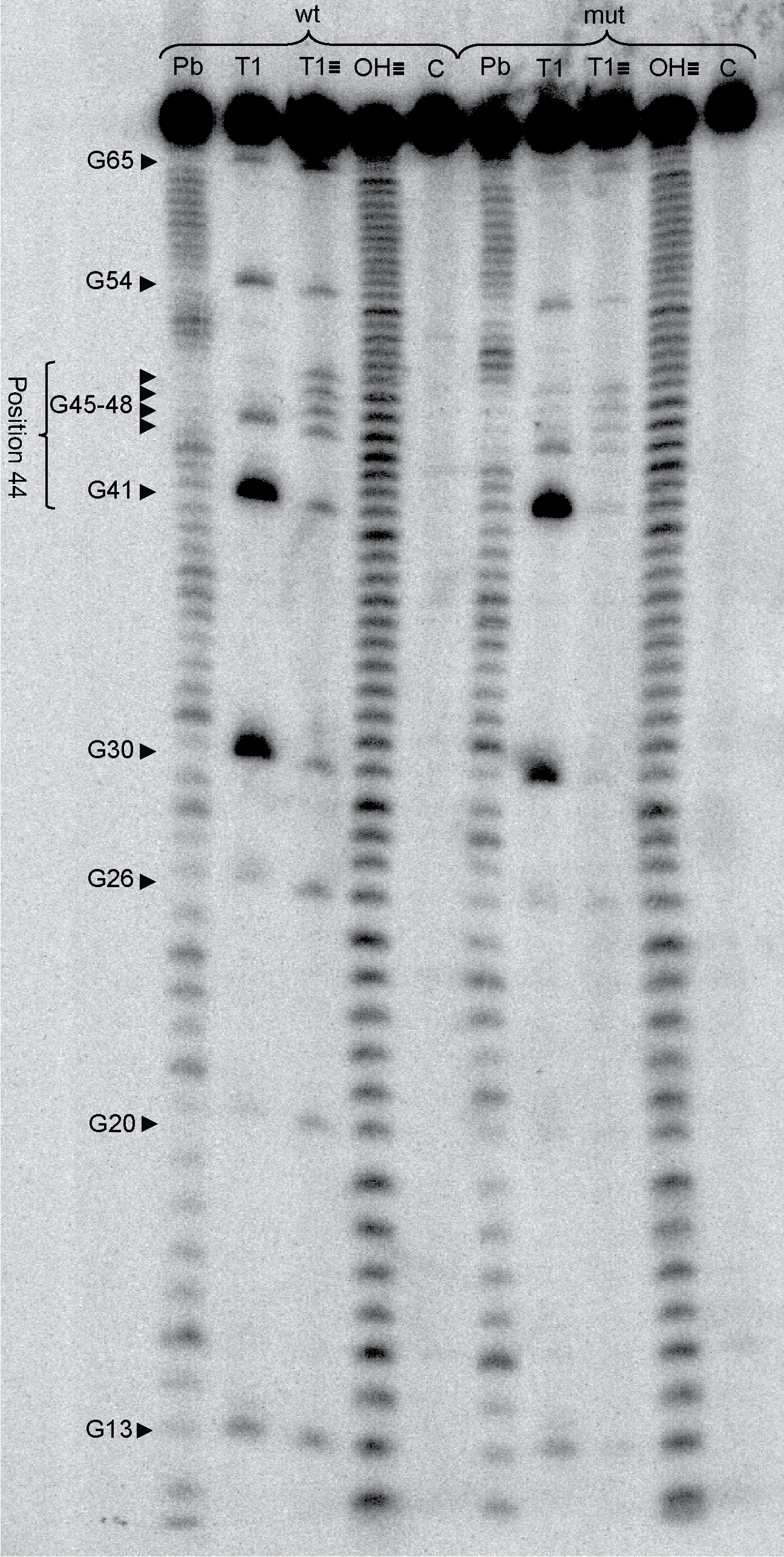

Supplement: Figure S2 — Structure probing of tRNATyr. Transfer RNATyr was in vitro transcribed, labeled and cleaved with RNase T1 or Pb2+. By comparing lanes from wt and mutant there is no obvious alteration in the structure of the mutant tRNATyr. (4.46 MB TIF) [file pgen.1000499.s002.tif]
