# Supplementary material for: Sensory Ataxic Neuropathy in Golden Retriever Dogs Is Caused by a Deletion in the Mitochondrial tRNATyr Gene
Source: PLoS Genet. 2009 May 29;5(5):e1000499. doi: 10.1371/journal.pgen.1000499 (PMC2683749; doi:10.1371/journal.pgen.1000499)
Supplement: Table S1 — Overview of heteroplasmy in blood and muscle, and morphological and biochemical examination in the five dog pairs tested. (0.05 MB DOC) [file pgen.1000499.s003.doc]

**Table S1: Overview of heteroplasmy in blood and muscle, and morphological and biochemical examination in the five dog pairs tested.** n.d. = not determined

| **Dog** | **Heteroplasmy in blood** | **Heteroplasmy in muscle** | **Histochemistry** | **Electron microscopy** |
| --- | --- | --- | --- | --- |
| Control 1 | 100% wt | n.d. | Normal | Normal |
| Control 2 | 100% wt | n.d. | Normal | Normal |
| Control 3 | 100% wt | 100% wt | Abnormal | Normal |
| Control 4 | 100% wt | n.d. | Normal | Normal |
| Control 5 | 100% wt | n.d. | Normal | Normal |
|  |  |  |  |  |
| Affected 1 | 3.2% wt | n.d. | Abnormal | Normal |
| Affected 2 | 9.2% wt | n.d. | Abnormal | Normal |
| Affected 3 | 4.0% wt | 0.9% wt | Normal | Paracrystalline inclusions |
| Affected 4 | 1.5% wt | n.d. | Abnormal | Normal |
| Affected 5 | 3.0% wt | n.d. | Abnormal | Normal |

| **Dog** | **Citrate synthase activity (mmol/min/kg muscle)** |  | **Respiratory chain enzyme activities (Units/unit CS)** | | | | |  | **Mitochondrial ATP production rates (units/unit CS)** | | | | |
| --- | --- | --- | --- | --- | --- | --- | --- | --- | --- | --- | --- | --- | --- |
|  |  |  | **NQR (I)** | **NCR (I/III)** | **SDH (II)** | **SCR (II/III)** | **COX (IV)** |  | **Glu+Succ** | **TMPD+Asc** | **Pyr+Mal** | **PalCar+Mal** | **Succ+Rot** |
| Control 1 | 36 |  | 0.191 | 2.07 | 0.35 | 0.73 | 2.56 |  | 0.25 | 0.27 | 0.16 | 0.12 | 0.065 |
| Control 2 | 43 |  | 0.137 | 1.57 | 0.35 | 0.77 | 2.96 |  | 0.18 | 0.17 | 0.10 | 0.07 | 0.053 |
| Control 3 | 72 |  | 0.055 | 1.08 | 0.37 | 0.79 | 2.66 |  | 0.16 | 0.24 | 0.14 | 0.10 | 0.047 |
| Control 4 | 81 |  | 0.051 | 0.60 | 0.33 | 0.67 | 2.73 |  | 0.17 | 0.23 | 0.15 | 0.11 | 0.046 |
| Control 5 | 71 |  | 0.203 | 1.72 | 0.39 | 0.85 | 3.14 |  | 0.12 | 0.19 | 0.13 | 0.11 | 0.036 |
|  |  |  |  |  |  |  |  |  |  |  |  |  |  |
| Affected 1 | 106 |  | 0.014 | 0.21 | 0.24 | 0.36 | 0.41 |  | 0.08 | 0.08 | 0.05 | 0.08 | 0.037 |
| Affected 2 | 107 |  | 0.044 | 0.91 | 0.30 | 0.59 | 1.53 |  | 0.12 | 0.10 | 0.08 | 0.07 | 0.033 |
| Affected 3 | 76 |  | 0.087 | 1.26 | 0.39 | 0.83 | 2.53 |  | 0.22 | 0.22 | 0.13 | 0.07 | 0.060 |
| Affected 4 | 77 |  | 0.051 | 0.44 | 0.28 | 0.47 | 0.69 |  | 0.10 | 0.09 | 0.09 | 0.09 | 0.039 |
| Affected 5 | 81 |  | 0.038 | 0.46 | 0.35 | 0.65 | 0.64 |  | 0.14 | 0.11 | 0.12 | 0.13 | 0.051 |
