# Supplementary material for: Sensory Ataxic Neuropathy in Golden Retriever Dogs Is Caused by a Deletion in the Mitochondrial tRNATyr Gene
Source: PLoS Genet. 2009 May 29;5(5):e1000499. doi: 10.1371/journal.pgen.1000499 (PMC2683749; doi:10.1371/journal.pgen.1000499)
Supplement: Table S2 — PCR and sequencing primers. (0.10 MB DOC) [file pgen.1000499.s004.doc]

| **Table S2: PCR and sequencing primers** |  |  |  |
| --- | --- | --- | --- |
| **A** mtDNA PCR primers* |  |  |  |
| **Sequence 5'-3'** | **Name** | **Start position (bp)** | **Prod. size (bp)** |
| GTCGCACGACTCCATAAACA | Pp1F | 50 | 1146 |
| CGCTCCAATTAAAATTTCTATCTCC | Pp1R | 1196 |  |
| TAAAGCGTCTGGCCTACACC | Pp2F | 1040 | 1215 |
| TCACCCCAACCTAAATTGCT | Pp2R | 2255 |  |
| GAGGCGGGAATACCACAATA | Pp3F | 2122 | 1286 |
| CGAAAGGACCTGCTGCATAC | Pp3R | 3408 |  |
| CCTGACCCCTAGCCATGATA | Pp4F | 3280 | 1210 |
| AGCGGCTATTCAGCCTATGT | Pp4R | 4490 |  |
| CGCACCTATATCCATCCTATATCA | Pp5F | 4322 | 1262 |
| CAAAGCCCCCAATTATGATG | Pp5R | 5584 |  |
| CTGCTTTGAGCCTCCTCATC | Pp6F | 5440 | 1246 |
| GCATCTGGGTAGTCAGAGTATCG | Pp6R | 6686 |  |
| TGCCCACTGATTCCCTTTAT | Pp7F | 6528 | 1280 |
| GGCATTTCATTAAGGAGAGGTT | Pp7R | 7808 |  |
| TGCGGATCTAACCACAGCTT | Pp8F | 7632 | 1259 |
| TTCCGTATCGTAGTCCTTTTTG | Pp8R | 8891 |  |
| GATTTCACTATAACTCAATAGCCCTAC | Pp9F | 8745 | 1272 |
| CCCTCTAAGCATAGTAGCGATGA | Pp9R | 10017 |  |
| GGGGCTAGAATGAACCGAAT | Pp10F | 9823 | 1282 |
| ACCGCTACGATAACCAGTGC | Pp10R | 11105 |  |
| CCCCTGACCAATTTCATAGC | Pp11F | 10957 | 1267 |
| GCCAACTCCTTCTCAACCAA | Pp11R | 12224 |  |
| CCCTTTTTGTCACGTGGTCT | Pp12F | 12058 | 1258 |
| TTTGAGTCCTTGTGAGGTAAGGT | Pp12R | 13316 |  |
| CGGAAGTGTATTTGCAGGGTA | Pp13F | 13157 | 1280 |
| TGCGTGCATATAGCGGATAA | Pp13R | 14437 |  |
| TGATGGAACTTCGGATCCTT | Pp14F | 14273 | 1168 |
| AATCTCAGCTTTGGGTGCTG | Pp14R | 15441 |  |
| GCGTTATCGAAAACAACCTTC | Pp15F | 15294 | 1625 |
| TGTGCTTGATACCTGCTCCTT | Pp15R | 189 |  |
| *PCRprimers: PpXF-PpXR | | | |

| **B** mtDNA sequencing primers (internal)^ |  |  |
| --- | --- | --- |
| **Sequence 5'-3'** | **Name** | **Start position (bp)** |
| GGGCTAAGCATAGTGGGGTA | S_Pp1R | 519 |
| AAGCCGTAAAAAGCTACAGTTATCA | Sp1F | 404 |
| GTGCTTCATGGCCCTATTCA | Sp1R | 898 |
| GTGGGAAGAAATGGGCTACA | S_Pp1F | 770 |
| TCCTGATTCTAAGAAGCTGTACCT | S_Pp2R | 1553 |
| GCCTAACGAGCCTGGTGATA | Sp2F | 1420 |
| GTTTGTGTTTGCCGAGTTCC | Sp2R | 1915 |
| CAGGAACGGATAGACCACTGA | S_Pp2F | 1772 |
| GGCCTTACATCCCTTGTCCT | S_Pp3R | 2582 |
| TGCAGCAGCTATTAAGGGTTC | Sp3F | 2448 |
| TGGGAGGGGAATTCACATAG | Sp3R | 3017 |
| CAATCGCAGACGCAGTAAAA | S_Pp3F | 2890 |
| TGGGATACTTGCGGTGATAA | S_Pp4R | 3692 |
| CCATTCATACCAGAACTCTACTCTATT | Sp4F | 3492 |
| CCTCTATGGCTCGTGGATTG | Sp4R | 4077 |
| CCCGTACTAATAAAACCCCCTA | S_Pp4F | 3906 |
| GCGGTGCTATATGTGAGTCG | S_Pp5R | 4816 |
| TATCTGGCTTCATCCCCAAA | Sp5F | 4684 |
| GCGGGAGAAGGTAGATTGAA | Sp5R | 5190 |
| ATCACCTCTAAGGGCTGCAA | S_Pp5F | 5030 |
| GCCAGTACAGGCAGGGATAG | S_Pp6R | 5957 |
| AGCATCCGTTGACCTTACAA | Sp6F | 5769 |
| TCCCGTTGGAATAGCGATAA | Sp6R | 6300 |
| AAAAGAGCCTTTCGGTTATATAGGA | S_Pp6F | 6141 |
| GAGGTTGCGTCCTGTAATCC | S_Pp7R | 7075 |
| GAAGGAATCGAACCCCCTAA | Sp7F | 6900 |
| CAGTATCATTGGTGGCCTATTG | Sp7R | 7351 |
| ACCCGCCATTATCCTAATCC | S_Pp7F | 7238 |
| AGCTCAGGTTCGTCCCTTTT | S_Pp8R | 8171 |
| TGCCCCCTCAATAATAGGTC | Sp8F | 7994 |
| GGTAGCCCCTCCAATCAAAT | Sp8R | 8498 |
| GCACACTTTCTACCCCAAGG | S_Pp8F | 8340 |
| CGTGTAATCCGTGAAATCCA | S_Pp9R | 9266 |
| CGCAAACATATACTTCAAGCCTTA | Sp9F | 9110 |
| TCGTAGGGGCTTGTCTTGTC | Sp9R | 9609 |
| TCCAGATCAACCTGGAAAGAA | S_Pp9F | 9473 |
| AAGGGGCAGAAGTCATGTTG | S_Pp10R | 10428 |
| TCCCCCTTACATGAATATCAAA | Sp10F | 10238 |
| GATCAGGAGTTTGGCAGAGG | Sp10R | 10775 |
| TGATTCCGACCCTGATCATT | S_Pp10F | 10580 |
| TTACCACGTTGTGTGGTGATT | S_Pp11R | 11456 |
| GCCTCACAAATCTGGCTCTC | Sp11F | 11291 |
| TCTGGAGTTGCACCAATTTTT | Sp11R | 11772 |
| TTTGCTTACCGAAAAAGTACTGC | S_Pp11F | 11641 |
| TGGATGAAAGCGGATAAGAA | S_Pp12R | 12575 |
| GCAGCTACCGGTAAATCTGCT | Sp12F | 12435 |
| ATGCGAGGCTTCCGATAATA | Sp12R | 12918 |
| CATCTGCACTCACGCATTTT | S_Pp12F | 12764 |
| TATGGGACTTGGTTGGTGGT | S_Pp13R | 13668 |
| AAAGGTCTCATCAAACTCTATTTCC | Sp13F | 13521 |
| ATTTATGGTGGGCTTGTGCT | Sp13R | 14034 |
| TCACACCTCAGGGTATTGCTC | S_Pp13F | 13864 |
| GGATCCGGTTTCGTGTAGAA | S_Pp14R | 14800 |
| GCCATCCCTTATATCGGAAC | Sp14F | 14639 |
| TGTGTGGAGGAGTGGAATGA | Sp14R | 15112 |
| AACTACACCCCTGCAAACC | S_Pp14F | 14948 |
| ACCAGATGCATGACACCACA | S_Pp15R | 15919 |
| AATCAGCCCATGATCACACA | Sp15F | 15876 |
| GGTTTGGCGGGACATAAATA | Sp15R | 16329 |
| CACGCGCGTAAGACATTAAG | S_Pp15F | 16234 |
| ^Sequencing primers: PpXF-S_PpXR, SpXF-SpXR, S_PpXF-PpXR | | |
