# Supplementary material for: Sensory Ataxic Neuropathy in Golden Retriever Dogs Is Caused by a Deletion in the Mitochondrial tRNATyr Gene
Source: PLoS Genet. 2009 May 29;5(5):e1000499. doi: 10.1371/journal.pgen.1000499 (PMC2683749; doi:10.1371/journal.pgen.1000499)
Supplement: Table S3 — Quantification primers. (0.03 MB DOC) [file pgen.1000499.s005.doc]

| **Table S3: Quantification primers** |  |  |
| --- | --- | --- |
| **A** Pyrosequencing |  |  |
| **Sequence 5'-3'** | **Name** | **Product size (bp)** |
| AAAATTCACCACGGAGCTT | Pyro_F | 94 |
| biotin-TGAACATAGGTAAAATGGCTGA | Pyro_R |  |
| GGCAAAAAGAGGACTTAAACC | Seq_del |  |
|  |  |  |
| **B** quantitative oligonucleotide ligation assay (qOLA) |  |  |
| **PCR primers** |  |  |
| **Sequence 5'-3'** | **Name** | **Product size (bp)** |
| GGCTGCTTCTTTGAATTTGC | qOLA_F_PCR | 222 |
| AGGCTCAAAGCAGTGCCTAC | qOLA_R_PCR |  |
| **Ligation primers** |  |  |
| **Sequence 5'-3'** | **Name** |  |
| GGCAAAAAGAGGACTTAAACCCC | LigR´ |  |
| Phos-TATCTTTAGATTTACAGTCTAATGCAAAAAAAAAA | LigF´1 |  |
| Phos-ATCTTTAGATTTACAGTCTAATGCAAAAAAAAAAAAAAA | LigF´2 |  |
|  |  |  |
