# Supplementary material for: Sensory Ataxic Neuropathy in Golden Retriever Dogs Is Caused by a Deletion in the Mitochondrial tRNATyr Gene
Source: PLoS Genet. 2009 May 29;5(5):e1000499. doi: 10.1371/journal.pgen.1000499 (PMC2683749; doi:10.1371/journal.pgen.1000499)
Supplement: Table S4 — Northern probes. (0.02 MB DOC) [file pgen.1000499.s006.doc]

| **Table S4: Northern probes** |  |  |
| --- | --- | --- |
| **Sequence 5'-3'** | **Name** | **tRNA size (bp)** |
| AAGAATTCAAAGATCTTCGTGCTACCAA | Probe_Gln | 74 |
| GTCTAATGCTTTTATCAGCCATTTTACC | Probe_Tyr(28) | 68 |
| GGCTGCTTCTTTGAATTTGCAATTC | 2_Cys_probe | 68 |
