# Supplementary material for: Sensory Ataxic Neuropathy in Golden Retriever Dogs Is Caused by a Deletion in the Mitochondrial tRNATyr Gene
Source: PLoS Genet. 2009 May 29;5(5):e1000499. doi: 10.1371/journal.pgen.1000499 (PMC2683749; doi:10.1371/journal.pgen.1000499)
Supplement: Table S5 — Structure probing sequences. (0.03 MB DOC) [file pgen.1000499.s007.doc]

| **Table S5: Structure probing sequences** |  |  |
| --- | --- | --- |
| **A** PCR primers |  |  |
| **Sequence 5'-3'** | **Name** | **Prod. size (bp)** |
| TTTGAATTCGAAATTAATACGACTCACTATAGGTAAAATGGCTGATAAAAGCATTAG | T7+Tyr_F_primer | 99 |
| TGGCAAAAAGAGGACTTAAAC | Tyr_R_primer |  |
|  |  |  |
| **B** Sequencing primers |  |  |
| **Sequence 5'-3'** | **Name** |  |
| TGAATTCGAAATTAATACGACTCACT | tRNA_sek_Fwd |  |
| TGGCAAAAAGAGGACTTAAAC | Tyr_R_primer |  |
