# Supplementary material for: Sensory Ataxic Neuropathy in Golden Retriever Dogs Is Caused by a Deletion in the Mitochondrial tRNATyr Gene
Source: PLoS Genet. 2009 May 29;5(5):e1000499. doi: 10.1371/journal.pgen.1000499 (PMC2683749; doi:10.1371/journal.pgen.1000499)
Supplement: Table S6 — Preparation of OH-ladder, T1-ladder, Control, T1 (0.01 U), Pb2+ (25 mM). (0.04 MB DOC) [file pgen.1000499.s008.doc]

| **Table S6: Preparation of OH-ladder, T1-ladder, Control, T1 (0.01U), Pb2+ (25mM)** | | |
| --- | --- | --- |
| **A** Preparation of OH-ladder | | |
| 1 ul labeled RNA |  |  |
| 9 ul alkaline buffer |  |  |
| 5 min at 90°C |  |  |
| 10 ul loading buffer |  |  |
| Put on ice |  |  |
|  | | |
| **B** Preparation of T1-ladder | | |
| 1 ul labeled RNA |  |  |
| 9 ul sequencing buffer |  |  |
| 1 min at 90°C |  |  |
| 1 ul T1 (0.1U) |  |  |
| 5 min at 37°C |  |  |
| 9 ul loading buffer |  |  |
| Put on ice |  |  |

| **C** Preparation of hotmix and cleavage reaction | | | |
| --- | --- | --- | --- |
| **Hotmix (per rxn)** |  |  |  |
| 1 ul labeled RNA |  |  |  |
| 4 ul dH2O |  |  |  |
| 1 min 90°C |  |  |  |
| Put on ice |  |  |  |
| 2 ul 5xTMN |  |  |  |
|  |  |  |  |
| **Cleavage reaction** | Control | T1 (0.01U)* | Pb (25mM)^ |
| Hotmix | 7 ul | 7 ul | 7 ul |
| dH2O | 3 ul | 1 ul | 1 ul |
| Enzyme/Pb | 0 ul | 2 ul | 2 ul |
| *Enzyme cleavage: Mix hotmix and dH2O. Incubate 15 min at 37°C. Add enzyme, incubate 5 min at 37°C and stop reaction by adding 5 ul 0.1M EDTA, put on ice. | | | |
| ^Lead cleavage: Mix hotmix and dH2O, incubate 15 min at 37°C. Add lead and incubate 1 min at 37°C and stop reaction by adding 5 ul 0.1M EDTA, put on ice. | | | |
