# Supplementary material for: Sensory Ataxic Neuropathy in Golden Retriever Dogs Is Caused by a Deletion in the Mitochondrial tRNATyr Gene
Source: PLoS Genet. 2009 May 29;5(5):e1000499. doi: 10.1371/journal.pgen.1000499 (PMC2683749; doi:10.1371/journal.pgen.1000499)
Supplement: Text S1 — Evaluation of quantification methods - pyrosequencing and quantitative oligonucleotide ligation assay. (0.02 MB DOC) [file pgen.1000499.s009.doc]

Supporting information: Baranowska et al.

**Text S1**

**Evaluation of Quantification Methods – Pyrosequencing and Quantitative Oligonucleotide Ligation Assay**

Results

- First we evaluated the resolution of both pyrosequencing and quantitative oligonucleotide ligation assay (qOLA) by creating two dilution series. One dilution series ranged from 0-100% wt sequence (T; wide) and the other one from 0-10% T (narrow). By comparing the resolution of Fig. S1 A with C it is apparent that the sensitivity of qOLA is superior (see 0 and 100% T). This is further supported by the narrow dilution serie (Fig. S1 B and D) where qOLA dilution series show linearity whereas pyrosequencing does not. Thus, our data suggest that qOLA gives a better resolution than pyrosequencing, and we therefore analyzed the heteroplasmy using qOLA.

Methods

The qOLA method is described in the main paper, whereas pyrosequencing was performed using the Pyro Gold chemistry (Biotage, Uppsala, Sweden). The qOLA and pyrosequencing primers are listed in Table S3. A distant relative showing approximatelly 50% wt and 50% mutant sequence (Fig. 2B) was PCR amplified using the Pp5F and Pp5R primers (Table S2 A) and cloned by TOPO TA cloning kit (Invitrogen, Carlsbad, CA). Single colonies were chosen and pyrosequenced. One wt and one mutant colony were grown, purified, and diluted to the same concentration. The two variants were mixed into the wide and narrow dilution series. The dilution series were then used to evaluate pyrosequencing and qOLA methods.
